# Proteomic analysis of Atg8-dependent recruitment of phagosomal proteins in the enteric protozoan parasite *Entamoeba histolytica*

**DOI:** 10.3389/fcimb.2022.961645

**Published:** 2022-10-03

**Authors:** Kumiko Nakada-Tsukui, Natsuki Watanabe, Kumiko Shibata, Ratna Wahyuni, Eri Miyamoto, Tomoyoshi Nozaki

**Affiliations:** ^1^ Department of Parasitology, National Institute of Infectious Diseases, Tokyo, Japan; ^2^ Department of Biomedical Chemistry, Graduate School of Medicine, The University of Tokyo, Tokyo, Japan

**Keywords:** autophagy, Atg8, *Entamoeba histolytica*, phagosome, proteome, gene silencing, lysosome, endoplasmic reticulum

## Abstract

Autophagy is one of the bulk degradation systems and is conserved throughout eukaryotes. In the enteric protozoan parasite *Entamoeba histolytica*, the causative agent of human amebiasis, Atg8 is not exclusively involved in autophagy per se but also in other membrane traffic-related pathways such as phagosome biogenesis. We previously reported that repression of *atg8* gene expression by antisense small RNA-mediated transcriptional gene silencing (gs) resulted in growth retardation, delayed endocytosis, and reduced acidification of endosomes and phagosomes. In this study, to better understand the role of Atg8 in phagocytosis and trogocytosis, we conducted a comparative proteomic analysis of phagosomes isolated from wild type and *atg8*-gs strains. We found that 127 and 107 proteins were detected >1.5-fold less or more abundantly, respectively, in phagosomes isolated from *the atg8*-gs strain, compared to the control strain. Among 127 proteins whose abundance was reduced in phagosomes from *atg8*-gs, a panel of proteins related to fatty acid metabolism, phagocytosis, and endoplasmic reticulum (ER) homeostasis was identified. Various lysosomal hydrolases and their receptors also tend to be excluded from phagosomes by *atg8*-gs, reinforcing the notion that Atg8 is involved in phagosomal acidification and digestion. On the contrary, among 107 proteins whose abundance increased in phagosomes from *atg8*-gs strain, ribosome-related proteins and metabolite interconversion enzymes are enriched. We further investigated the localization of several representative proteins, including adenylyl cyclase-associated protein and plasma membrane calcium pump, both of which were demonstrated to be recruited to phagosomes and trogosomes *via* an Atg8-dependent mechanism. Taken together, our study has provided the basis of the phagosome proteome to further elucidate molecular events in the Atg8-dependent regulatory network of phagosome/trogosome biogenesis in *E. histolytica*.

## Introduction

Autophagy is widely conserved among eukaryotes. Although it was initially found as a bulk degradation system to survive amino acid starvation (Tsukada and Ohsumi, 1993; Nakatogawa, 2020), diverse biological roles of autophagy such as innate immunity, antigen presentation, and protein secretion have been established in model organisms (Nakagawa et al., 2004; Zhang et al., 2015; Loi et al., 2016). The identification of autophagy-related (Atg) proteins and their interacting proteins enables conducting gene surveys for non-model organisms. Such a genome-wide survey revealed the high conservation of two ubiquitin-like conjugation pathways, with Atg5-12 and Atg8 being principal components (Duszenko et al., 2011; Sakamoto et al., 2021; Zhang et al., 2021). Atg8 and Atg12 are ubiquitin-like modifiers and are involved in the conjugation with phosphatidylethanolamine (PE) and Atg5, respectively. The conjugation is mediated by a panel of enzymes that are similar to the authentic ubiquitin conjugation system: Atg7 as E1, and Atg3/Atg10 as E2 for Atg8 or Atg12, respectively. Although the pivotal role of Atg8 in autophagy is well understood and Atg8 is used as an authentic marker for autophagy, the general role of Atg8 in non-model organisms is not fully elucidated (Karpiyevich and Artavanis-Tsakonas, 2020). In the course of autophagy, cytosolic Atg8 and its accessory proteins are accumulated at the isolation membrane (also called the phagophore) to conjugate Atg8 to PE for membrane insertion. After the completion of autophagy, PE-modified Atg8 is removed by Atg4 protease for the next round of membrane interaction (Nair et al., 2012). Initially, Atg8 was thought to be engaged with the extension of the isolation membrane. However, the premise is now dismissed and, instead, the process is believed to be cooperatively regulated by Atg9 and Atg2 (Nguyen et al., 2016; Matoba et al., 2020). The discovery of Atg8-interacting motifs (AIRs) (LC3-interacting regions, LIRs in mammals) has enabled the prediction of the proteins that interact with Atg8 *via* AIR. Various AIR-containing proteins have been identified including autophagy cargos and adaptor proteins involved in vesicular transport and fission (Birgisdottir et al., 2013). These predicted Atg8-binding proteins are known to bind to Atg8 on both the inner and outer membranes, select specific cargos, and facilitate autophagosome degradation (Pankiv et al., 2010; Fu et al., 2014; Rogov et al., 2014; McEwan et al., 2015; Søreng et al., 2018; Kirkin and Rogov, 2019). Atg8-PE is also implicated in membrane tethering, hemifusion and fusion, deformation, and out-budding (Nakatogawa et al., 2007; Maruyama et al., 2021), thus implying that it plays a central role as a direct regulator in a variety of membrane dynamics.

*Entamoeba histolytica* is a protozoan parasite and the causative agent of human amebiasis. This parasite has two life cycle stages, the dormant cyst, and the motile, proliferative, and disease-causing trophozoite. Cysts excreted into the stool are responsible for fecal-oral transmission. Ingested cysts can pass through the stomach and excyst to form trophozoites and colonize the colon. Trophozoites are responsible for clinical manifestations. The major symptoms of intestinal amebiasis are diarrhea, colitis, and dysentery. Around 10% of intestinal amebiasis patients further developed extraintestinal amebiasis, with the most common presentation being liver abscesses. Several major virulence mechanisms of amebiasis known up to date include adhesion to host cells and tissues, proteolysis by proteases to destroy host extracellular matrix and tissues, evasion from host immune response (Petri et al., 2002; Thibeaux et al., 2012; Thibeaux et al., 2014; Nakada-Tsukui and Nozaki, 2016). Phagocytosis and trogocytosis are also implemented as part of important virulence mechanisms. Phagocytosis and trogocytosis differ in that prey, in the case of dead mammalian cells, undeformable bacteria, and fungi, is internalized by a single bite in phagocytosis and, in the case of live mammalian cells, prey is partially and gradually internalized by multiple bites (nibbling). Phagocytosis and trogocytosis are involved in nutrient acquisition, removal of immune cells, and invasion of host tissue for colonization (Ralston et al., 2014; Bettadapur et al., 2020; Nakada-Tsukui and Nozaki, 2021).

Autophagy plays an important role in lysosome maturation in trophozoites and encystation (trophozoite-to-cyst differentiation) in *Entamoeba* (Picazarri et al., 2008; Picazarri et al., 2015). Atg8 is constitutively expressed in trophozoites and is involved in lysosome acidification, degradation of its content, and growth (Picazarri et al., 2015). In *E. invadens*, massive upregulation of Atg8 occurs during encystation under low glucose and low osmolarity conditions (Picazarri et al., 2008). Autophagy is also operated in stage conversion of other protozoa including *Acanthamoeba* and *Leishmania* (Besteiro et al., 2006; Moon et al., 2013). Two conjugation pathways including Atg8 and Atg5-12 are well conserved in *Entamoeba* (Picazarri et al., 2008; Picazarri et al., 2015). In contrast to model organisms, *E. histolytica* Atg8 is lipidated even in nutrient-rich conditions and, thus, the presence of nutrient-deprivation induced autophagy has not been established. Professional phagocytes such as antigen-presenting cells from mammals and single-cell organisms such as *Entamoeba*, capable of ingestion of particles of various sizes, show two modes of ingestion: trogocytosis and phagocytosis. In both trogocytosis and phagocytosis of *Entamoeba*, Atg8 is immediately recruited to the trogocytic (and phagocytic) cup, the bottom portion of the concave formed from the plasma membrane (Picazarri et al., 2015). It was also shown that repression of *atg8* gene expression by small antisense RNA-mediated transcriptional gene silencing caused a reduction in phagosome acidification (Picazarri et al., 2015). These findings strongly support the key role of Atg8 in the formation and maturation of phago- and trogosomes during the internalization of the prey by this parasites.

In this study, to better understand the molecular basis of how Atg8 is involved in phagocytosis and trogocytosis and subsequent maturation of phago/trogosomes, we conducted the phagosome proteome analysis. We exploited a comparative proteomic analysis of phagosomes isolated from *atg8*-gene silenced (*atg8*-gs) and parental strains using human serum-coated paramagnetic beads. We assumed, by analogy to model organisms, that Atg8 directly or indirectly recruits other proteins involved in phagosome formation and maturation. In such a case, *atg8*-gs phagosomes should in theory show changes in the recruitment of phagosome-associated proteins involved in phagosome formation and maturation. We identified 127 and 107 proteins whose abundance decreased or increased in phagosomes isolated from *atg8*-silenced strain relative to control. We categorized and analyzed them based on gene ontology (GO). We found that the abundance of the proteins involved in phagocytosis, fatty acid metabolism, and ER-related proteins was reduced by *atg8* gene silencing. On the contrary, some groups of proteins were found enriched in phagosomes from the *atg8-*gs strain, including ribosome-related proteins and cytoplasmic metabolism-related enzymes. We further confirmed the localization of several representative proteins which were less abundant in phagosomes from the *atg8*-gs strain, including adenylyl cyclase associated protein (CAP) and plasma membrane calcium pump (PMCA). Intracellular localization of these proteins validated their recruitment *via* an Atg8-dependent mechanism.

## Materials and methods

### Cells, culture, and reagents

*E. histolytica* strain in which the *atg8* gene was silenced (*atg8*-gs) and the control strain (mock vector transfected) were produced from the G3 strain, as described previously (Picazarri et al., 2015). Trophozoites of these transformants were maintained axenically in Diamond’s BI-S-33 medium (BIS) (Diamond et al., 1978) supplemented with 10 μg/mL Geneticin at 35.5˚C. Chinese hamster ovary (CHO) cells were maintained in F12 medium (Sigma-Aldrich, St Louis, MO) supplemented with 10% fetal calf serum (Sigma-Aldrich, St Louis, MO) at 37˚C with 5% CO_2_. Paramagnetic Dynabeads, dithiobis succinimidyl propionate (DSP), and OPTI‐MEM I medium were purchased from Thermo Fisher Scientific (Waltham, MA). A complete mini mix was purchased from Roche (Basel, Switzerland). Human serum was purchased from Sigma-Aldrich (St Louis, MO). Anti-HA (11MO), anti-Myc (9E10), and anti-FLAG (clone M2) monoclonal antibodies were purchased from Covance (Princeton, NJ) and Sigma-Aldrich. The production of rabbit polyclonal antibodies against EhAtg8, EhCS1, and EhCP-A5 were previously described (Nozaki et al., 1999; Picazarri et al., 2008; Nakada-Tsukui et al., 2012). All chemicals of analytical grade were purchased form Sigma-Aldrich unless otherwise stated.

### Phagosome purification

Phagosomes were purified as previously described (Watanabe et al., 2020). Briefly, Paramagnetic Dynabeads of 2.8‐μm diameter (catalog number 14203) were incubated with human serum for 16 hr at 4°C, washed, and resuspended in transfection medium (OPTI‐MEM I medium, adjusted to pH 6.8, supplemented with 5 mg/ml L-cysteine and 1 mg/ml ascorbic acid). Approximately 2.4 × 10^7^ trophozoites were seeded to each well on a six‐well plate (Corning, New York, USA) and incubated at 35.5°C for 30 min to allow attachment to the surface of the well. After the medium was removed, a 2‐ml transfection medium containing approximately 1 × 10^7^ human serum‐coated beads was added to each well (at the ratio of 10 beads per cell). The plate was centrifuged at 100 × g at room temperature for 5 min to allow sedimentation of beads on the amebae. OPTI‐MEM was carefully removed to eliminate uningested beads, and 3 ml of warm BIS was added. The plate was further incubated at 35.5°C for 30 min. After incubation, the BIS medium was replaced with 3 ml of cold phosphate buffer saline (PBS), pH 7.4, and the plate was placed on ice for 10 min to detach the amoebae. The amoebae were washed twice with cold PBS, followed by centrifugation at 800 × g at 4°C for 3 min to remove the supernatant. The amoebae were finally resuspended in 500 μl of PBS containing 0.8 mg/ml DSP. The mixture was incubated on a rotator (10 rpm) at 4°C for 30 min. The suspension was subsequently mixed and incubated with 50 μl of 1 M Tris-HCl, pH 7.5 at 4°C for 10 min to quench the reaction. After the amoebae were washed with homogenization buffer (250 mM sucrose, 3 mM imidazole, in PBS, pH 7.4, and complete mini), amoebae were mechanically homogenized by applying 25–70 strokes of a Dounce homogenizer. The homogenate was centrifuged at 800 × g at 4°C for 3 min to remove unbroken cells. Bead‐containing phagosomes were concentrated from the lysates by paramagnetic separation on PureProteome Magnetic Stand (Merck) according to the manufacturer’s protocol. The phagosomes were washed five times with 500 μl of homogenization buffer containing 50 μg/ml of E‐64, and complete mini and lysed with 50 μl of lysis buffer (50 mM Tris HCl, pH 7.5, 150 mM NaCl, 1% Triton X‐100, containing 50 μg/ml of E‐64, and complete mini mix).

### Immunoblot analysis

Approximately 2 μg of isolated phagosomes were resuspended in SDS‐PAGE sample buffer (0.25M Tris‐HCl, pH 6.8, 8% SDS, 8% 2‐mercaptoethanol, 40% glycerol, 0.004% bromophenol blue), boiled for 5 min, and subjected to SDS‐PAGE. To confirm protein expression in trophozoites, approximately 1 × 10^5^ trophozoites were harvested in the exponential growth phase, washed twice with PBS, pH 7.4, and resuspended in 50 μL of lysis buffer. Approximately 20 μg of the total cell lysates were separated on 12% SDS‐polyacrylamide gels and subsequently electrotransferred onto nitrocellulose membranes. The membranes were incubated with 5% non‐fat dried milk in TBS‐T (50 mM Tris‐HCl, pH 8.0, 150 mM NaCl, and 0.05% Tween‐20) for 30 min. The proteins were reacted with mouse monoclonal antibodies specific for HA (with the dilution of 1:1000), Myc (1:1000), or FLAG (1:1000), or polyclonal rabbit antisera against EhCP‐A5 (1:1000) and CS1 (1:1000), at 4°C overnight. After the membranes were washed three times with TBS‐T, they were reacted with horseradish peroxidase (HRP)-conjugated anti-mouse or anti-rabbit IgG antiserum (1:6000 or 1:8000, respectively) at room temperature for 1 hr. After washing with TBS‐T three times, the specific proteins were visualized by chemiluminescence detection using Immobilon Western Chemiluminescent HRP Substrate (Millipore Corporation, MA, USA) according to the manufacturer’s protocol. The mean intensity (the total intensity per the area) of the protein bands was quantified using ‘measure’ tool in Image J software.

### Mass spectrometric analysis of phagosomes

The isolated phagosomes were briefly electrophoresed on SDS–PAGE to allow entry of proteins into the gel, visualized by silver stain, and the bands containing whole mixture were excised and subjected to LC–MS/MS analysis. The LC–MS/MS analysis was performed at W. M. Keck Biomedical Mass Spectrometry Laboratory, University of Virginia, USA. The gel pieces from the band were transferred to a siliconized tube and washed in 200 µL 50% methanol. The gel pieces were dehydrated in acetonitrile, rehydrated in 30 µL of 10 mM dithiothreitol in 0.1 M ammonium bicarbonate and reduced at room temperature for 0.5 h. The DTT solution was removed and the sample alkylated in 30 µL 50 mM iodoacetamide in 0.1 M ammonium bicarbonate at room temperature for 0.5 h. The reagent was removed and the gel pieces dehydrated in 100 µL acetonitrile. The acetonitrile was removed and the gel pieces rehydrated in 100 µL 0.1 M ammonium bicarbonate. The pieces were dehydrated in 100 µL acetonitrile, the acetonitrile removed and the pieces completely dried by vacuum centrifugation. The gel pieces were rehydrated in 20 ng/µL trypsin in 50 mM ammonium bicarbonate on ice for 30 min. Any excess enzyme solution was removed and 20 µL 50 mM ammonium bicarbonate added. The sample was digested overnight at 37˚C and the peptides formed extracted from the polyacrylamide in a 100 µL aliquot of 50% acetonitrile/5% formic acid. This extract was evaporated to 15 µL for MS analysis. The LC-MS system consisted of a Thermo Electron Velos Orbitrap ETD mass spectrometer system with a Protana nanospray ion source interfaced to a self-packed 8 cm x 75 um id Phenomenex Jupiter 10 um C18 reversed-phase capillary column. 7 µL of the extract was injected and the peptides eluted from the column by an acetonitrile/0.1 M acetic acid gradient at a flow rate of 0.5 µL/min over 1.2 hours. The nanospray ion source was operated at 2.5 kV. The digest was analyzed using the rapid switching capability of the instrument acquiring a full scan mass spectrum to determine peptide molecular weights followed by product ion spectra (20) to determine amino acid sequence in sequential scans. This mode of analysis produces approximately 40000 MS/MS spectra of ions ranging in abundance over several orders of magnitude. The mass spectrometry proteomics data have been deposited to the ProteomeXchange Consortium *via* the PRIDE (Perez-Riverol et al., 2022) partner repository with the dataset identifier PXD034376 and 10.6019/PXD034376.

### Data analysis of phagosome proteome

The proteome data were analyzed by the Sequest search (Eng et al., 1994) against *E. histolytica* proteome. The quantitative values (QV), normalized with unweighted spectrum counts, were used to estimate relative quantities of individual proteins in the samples. Phagosomal proteins that were detected more or less abundantly in *atg8*-gs strain than in the mock control were determined by the following criteria. Proteins that showed QV>2 in the control sample and showed >1.5-fold higher or <1.5-fold lower QV in *atg8*-gs strain, compared to the control mock strain were categorized to “phagosomal proteins that were detected in more or less, respectively, abundantly in *atg8*-gs strain than in the mock control”. The lists of detected proteins based on the criteria above from two independent experiments were merged and proteins that had been detected in both experiments were finally listed as differentially regulated proteins. The proteins were further analyzed using PANTHER 16.0 released protein class ontology (http://pantherdb.org/).

### Plasmid construction and transfection

Plasmids to express phagosome-associated proteins were constructed as follows. The full-length protein-coding region of adenylyl cyclase-associated protein (CAP, EHI_136150), plasma membrane calcium pump (PMCA, EHI_054853), EhSyntaxin B (EHI_021410), and Rab7G (EHI_187090) were PCR amplified with a SmaI site at the 5’ end and a XhoI site at the 3’ end. Then subcloned into a SmaI and an XhoI digested pEhExGFP vector (CAP) (Yousuf et al., 2010), pEhExHA vector (PMCA) (Nakada-Tsukui et al., 2012), pEhExMyc vector (EhSyntaxin B) (Nakada-Tsukui et al., 2012), and pEhExFLAG vector (EhRab7G) (Watanabe et al., 2020), respectively. The resultant plasmids were designated as pEhExGFP-CAP, pEhExHA-PMCA, EhExMyc-STX, and EhExFLAG-Rab7G, respectively. To establish the amoeba transformants, the trophozoites of HM‐1:IMSS cl6 were transfected with pEhExGFP-CAP, pEhExHA-PMCA, EhExMyc-STX, and EhExFLAG-Rab7G by lipofection as previously described (Nozaki et al., 1999). Geneticin was added at a concentration of 1 μg/ml 24 hr after transfection, then gradually raised until the geneticin concentration reached 10 μg/ml.

### Indirect immunofluorescence assay

An indirect immunofluorescence assay was performed as previously described (Nakada-Tsukui et al., 2012) with slight modifications. Briefly, trophozoites were transferred to an 8 mm round well on a slide glass and fixed with 3.7% paraformaldehyde in PBS at room temperature for 15 min. After washing three times with PBS, the cells were permeabilized with 0.2% saponin in PBS containing 1% bovine serum albumin (BSA) for 10 min and reacted with a primary antibody diluted at 1:300 (anti-Myc monoclonal antibody), 1:500 (anti-Atg8 polyclonal antibody), or 1:1000 (anti-HA and anti-FLAG monoclonal antibodies) in PBS containing 0.2% saponin and 1% BSA for 1h. After washing three times with PBS, the samples were then reacted with PBS containing 1% BSA and Alexa Fluor 488- or 568-conjugated anti-rabbit or anti-mouse secondary antibody (diluted at 1:1000) for 1 h. For phagosome staining, CHO cells were pre-stained with 10 μM CellTracker Blue (Molecular Probes, Eugene, OR, USA) for 30 min, harvested, and washed with BIS. Approximately 1.5×10^5^ amoeba cells were incubated with 3×10^5^ of CellTracker-stained CHO cells or 1.5×10^6^ of serum-coated beads, prepared as described above, for the indicated times. The samples were examined on a Carl-Zeiss LSM 780 confocal laser-scanning microscope. The resultant images were further analyzed using ZEN software (Carl Zeiss, Oberkochen, Germany).

## Results

### Defining Atg8-regulated phagosome-associated proteins based on changes in the abundance in phagosomes, caused by *atg8* gene silencing

**Figure 1:**
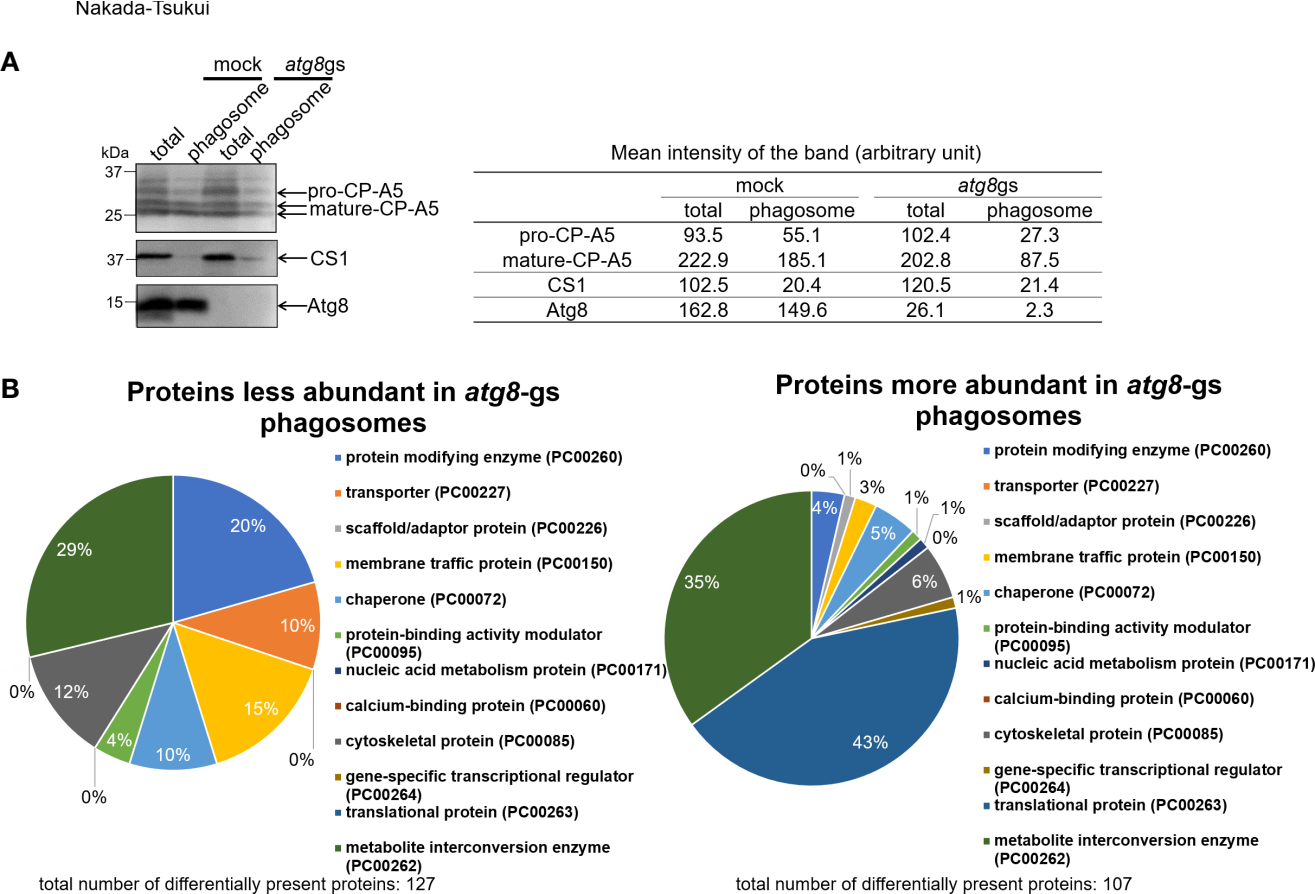
Evaluation of quality of purified phagosome and protein class distribution of less- and more- abundant proteins in *atg8*-gs strain. **(A)** Immunoblot analysis of total (total) and purified phagosome (phagosome) protein. *E histolytica* cell lysate from mock and *atg8*-gs strains are loaded, 20 μg for total and 2 μg for phagosome protein, were separated by SDS-PAGE. Each protein was detected with anti-EhCP-A5, anti-CS1, ad anti-Atg8 antisera, respectively. Quantification of the mean intensity (the total intensity per the area) of the bands is shown in the right table. **(B)** Protein class distribution of less- and more- abundant phagosomal proteins in *atg8-*gs.

To investigate how Atg8 regulates phagosome maturation, we conducted proteome analysis of phagosomes isolated from an *E. histolytica* strain in which *atg8* gene expression was silenced (*atg8* gene silenced, *atg8*-gs) and its mock control strain (transfected with psAP2-Gunma). Generation and phenotypic characterization of these strains were previously described (Picazarri et al., 2015); *atg8*-gs strain showed a delay in phagosome acidification compared to the control (Picazarri et al., 2015). In the present phagosome proteome analysis, human serum-coated paramagnetic beads were used as prey. We first verified the recruitment of Atg8 on the membrane of phagosomes containing beads at 30 min of coincubation (**Figure 1A**). We chose this time point as we were primarily interested in the early events of Atg8-dependent phagocytosis because the localization of Atg8 on the phago- and trogosomes peaks at 10 min and subsequently decreases (Picazarri et al., 2015). However, taking this very early time point (10 min) was technically difficult; in addition, the earliest time point used in the previous phagosome proteome studies for data comparison was 30 min. We first evaluated the purity of isolated phagosomes by immunoblot with anti-Atg8, cysteine protease-A5 (EhCP-A5, lysosomal maker), and cysteine synthase 1 (CS1, cytoplasmic marker) antisera. It has been previously shown that EhCP-A5 is synthesized as a pro form and proteolytically processed to be a mature form and present in both glycosylated and non-glycosylated forms in lysosomes (Nakada-Tsukui et al., 2012). As expected, the purified phagosome fraction contained mature EhCP-A5 and a less amount of pro-EhCP-A5 (**Figure 1A**). Only a negligible amount of CS1 was observed due to cross-contamination. Complete repression of *atg8* gene expression in the *atg8-*gs strain was also confirmed.

Phagosomes isolated from *atg8*-gs and mock strains in two independent experiments were analyzed by LC-MS/MS. A total of 996 and 1074 proteins, when data were combined from *atg8-*gs and control strains, were identified from phagosomes, in the 1^st^ and 2^nd^ experiments, respectively. Once the proteins with QVs < 2 in control or *atg8*-gs were removed, 438 and 615 proteins from control and 386 and 360 proteins from *atg8*-gs were defined as phagosomal proteins, respectively, in two experiments. Out of them, 338 and 259 proteins were shared in both experiments in control and *atg8*-gs strain, respectively (**Supplementary Tables 3**, **4**). Protein classification of 338 and 259 proteins by Protein ANalysis THrough Evolutionary Relationships (PANTHER) classification system by using PANTHER protein class annotation data set is shown (**Supplementary Figure 1**, **Supplementary Table 8**). In this annotation data set, protein class has less number of categories (around 250) than GO classification and is suitable for rough categorization of biologically significant groups of proteins (Thomas et al., 2003). It should be noted that the distribution of all phagosomal proteins also significantly differs between mock and *atg8*-gs strains.

To identify the phagosome-associated proteins less or more abundant in *atg8*-gs strain, compared to control strain, all detected proteins were divided into three groups: group 1, proteins detected >1.5 fold less abundantly in *atg8-*gs strain than in control with QV>2 in control; group 2, proteins detected >1.5 fold more abundantly in *atg8-*gs strain than in control with QV>2 (**Supplementary Tables 1**, **2**); and groups 3 containing the remaining proteins. We identified 127 and 107 proteins in groups 1 and 2, respectively. Subsequently, the proteins in each group were classified by the PANTHER classification system as explained above (**Figure 1B**). Out of 127 and 107 proteins in these two groups, described above, 105 and 100 proteins are registered in the PANTHER database. Among them, 73 and 83 proteins are categorized into 7 and 10 functional classes, respectively (**Figure 1B**). The distribution of the detected proteins to functional classes is largely different between groups 1 and 2. The proteins in group 1 (detected >1.5 fold less abundantly in phagosomes from *atg8-*gs strain) are evenly distributed among 7 functional classes, while the proteins in group 2 (detected >1.5 fold more abundantly in phagosomes from *atg8-*gs strain) were predominantly distributed to two dominant classes: translational protein (PC00263, 43%) and metabolite interconversion enzyme (PC00262, 35%).

### Proteins detected less abundantly in phagosomes from *atg8-*gs strain

**Figure 2:**
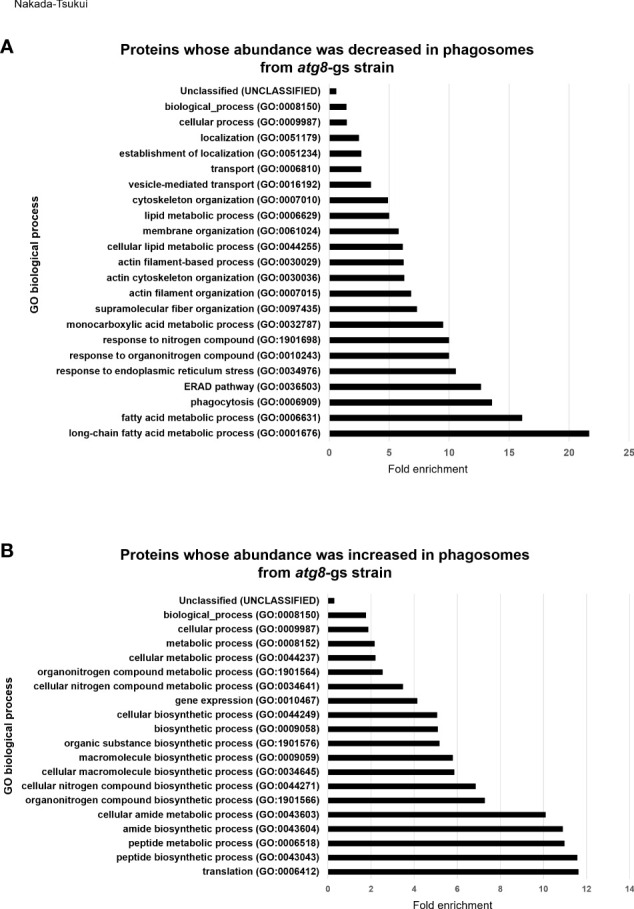
GO enrichment analysis of less- and more- abundant phagosomal proteins in *atg8*-gs by PANTHER. **(A)** Result of less abundant proteins in *atg8*-gs. All the significant result (false discovery rate, FDR, <0.05) was shown. **(B)** Result of more abundant proteins in *atg8*-gs. Only highly significant results (FDR < 1×10^10^) were shown. All the results with FDR < 0.05 was shown in **Supplementary Table 6**.

We subsequently conducted GO enrichment analysis of 127 proteins detected less (group 1) or 107 proteins detected more (group 2) abundantly in *atg8-*gs strain (**Supplementary Tables 1, 2**), by using PANTHER GO-Slim-data type [False discovery rate (FDR) <0.05]. This annotation data type consists of 3039 GO terms and is adequate to evaluate the enrichment of proteins that belong to particular biological functional classes (Gaudet et al., 2011). The proteins detected less abundantly in the *atg8-*gs strain are significantly enriched in 23 GO biological process annotations (**Figure 2A** and **Supplemental Table 5**). The two most enriched GO annotations are two metabolic processes related to fatty acid metabolism: long-chain fatty acid metabolic process (GO:0001676) and fatty acid metabolic process (GO:0006631). After these two metabolic processes, GO annotations such as proteins related to phagocytosis (GO: 0006909), ERAD pathway (GO:0036503), response to endoplasmic reticulum stress (GO:0034976), response to nitrogen compound (GO:1901698) are also enriched. The two most enriched GO annotations related to fatty acid metabolism contain 4 and 7 proteins, respectively. Similarly, three GO annotations after two metabolic processes, other than phagocytosis (GO: 0006909), contain 5 same proteins. Furthermore, 7 proteins are grouped into two GO annotations: monocarboxylic acid metabolic process, the 8^th^ ranked enriched annotation and fatty acid metabolic process. The top eight biological process annotations in which the proteins less abundant in *atg8-*gs phagosomes are enriched are mainly involved in fatty acid metabolism, phagocytosis, and endoplasmic reticulum (ER) homeostasis. Proteins categorized into phagocytosis include two Rab proteins (Rab7D, EHI_082070; Rab7G, EHI_187090), Rho GTPase (EHI_129750), calreticulin (EHI_136160), and coronin (EHI_082080). Following the top eight annotations, four annotations related to cytoskeleton regulation, such as supramolecular fiber organization (GO:0097435), actin filament organization (GO:0007015), actin cytoskeleton organization (GO:0030036), and actin filament-based process (GO:0030029), were identified. Eight proteins are mainly shared by four of these annotations.

### Proteins detected more abundantly in phagosomes from *atg8-*gs strain

We also conducted the same analysis for proteins detected more abundantly in phagosomes from the *atg8-*gs strain. Among 98 biological process annotations, 107 proteins are assigned, only annotations with higher significance, FDR less than 1x10^10^ are shown (**Figure 2B**) (those with FDR less than 0.05 are shown in **Supplementary Table 6**). All seven biological process annotations are shown in **Figure 2B**, commonly containing 35 identical proteins. These nine annotations are translation (GO:0006412), peptide biosynthetic process (GO:0043043), peptide metabolic process (GO:0006518), amide biosynthetic process (GO:0043604), cellular amide metabolic process (GO:0043603), cellular macromolecule biosynthetic process (GO:0034645), macromolecule biosynthetic process (GO:0009059), organonitrogen compound biosynthetic process (GO:1901566), and cellular nitrogen compound biosynthetic process (GO:0044271). The two latter annotations contain two or one additional protein, respectively. These shared proteins are all related to ribosomes. Following these annotations are the organic substance biosynthetic process (GO:1901576) and biosynthetic process (GO:0009058) all of which also contain 35 ribosomal proteins and 7 additional proteins (adenylate kinase, EHI_135470; aspartate-ammonia ligase, EHI_148470; cysteine synthase A, EHI_024230; geranylgeranyl pyrophosphate synthase, EHI_105060; inositol-3-phosphate synthase, EHI_070720; phosphoglycerate kinase, EHI_188180). In the cellular biosynthetic process (GO:0044249) 35 ribosomal proteins and 6 additional proteins are included. The list of these proteins is almost identical to the proteins listed in the organic substance biosynthetic process (GO:1901576) and the biosynthetic process (GO:0009058), with a single exception of phosphoglycerate kinase, EHI_188180. These results strongly suggest that there is a significant enrichment of ribosomal proteins in *atg8*-gs phagosomes.

### Immunolocalization of Atg8-regulated phagosome protein

**Figure 3:**
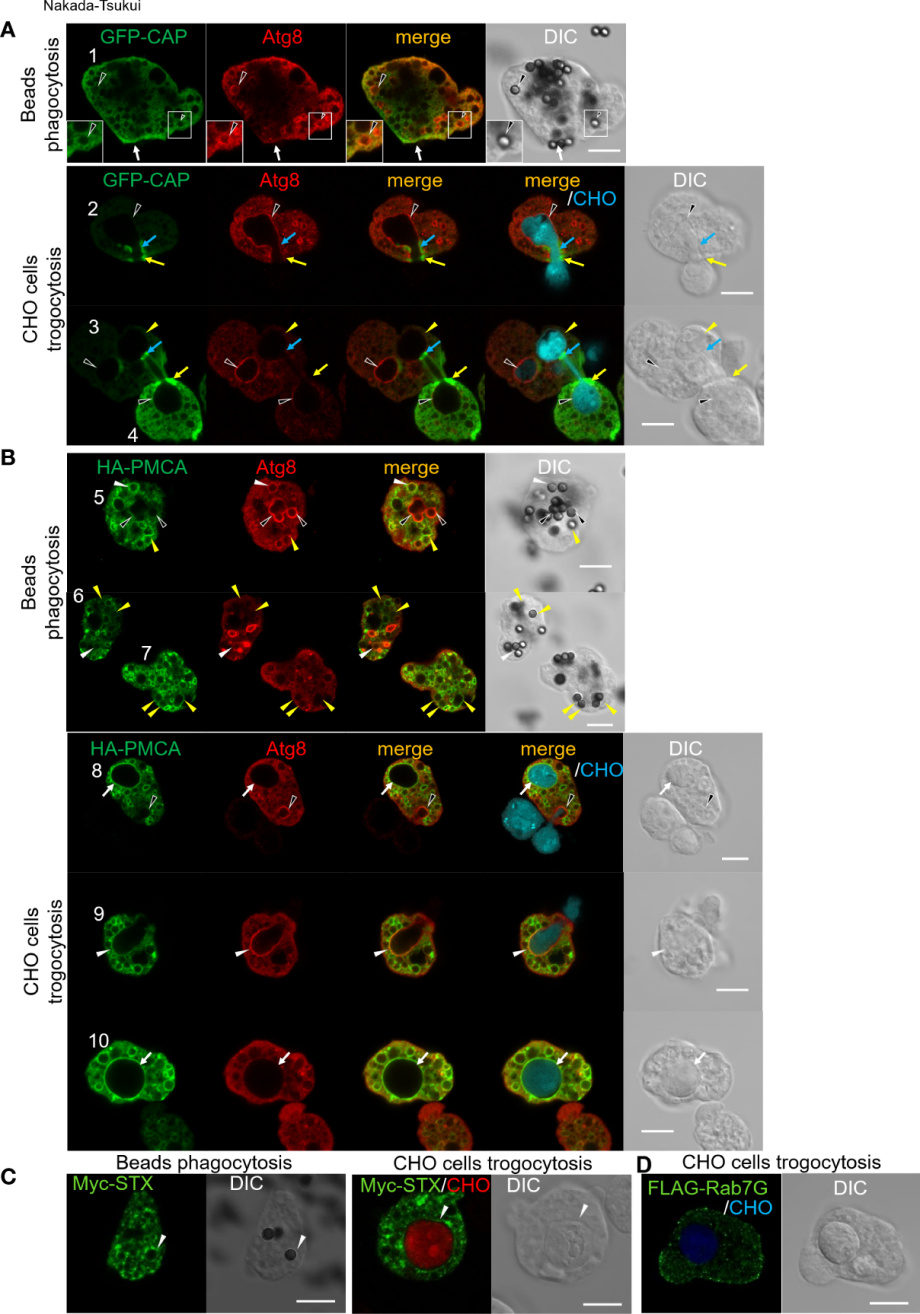
Subcellular localization of newly identified phagosomal proteins. Subcellular localization of tag-fused CAP **(A)**, PMCA **(B)**, STX **(C)**, and Rab7G **(D)** were shown in green (GFP or AlexaFluor 488). **(A)** GFP-CAP expressing *E histolytica* trophozoites were co-incubated with human serum coated beads for 30 min (upper panels) or CellTracker Blue-stained live CHO cells for 30 min (lower panels). Cells were fixed and localization of self-fluorescence of GFP and anti-Atg8 antibody stained Atg8 (Alexa Fluor 568) were shown. Black arrowheads, CAP negative Atg8 positive phagosome/trogosome; yellow arrowheads, CAP and Atg8 double-negative trogosome; white arrow, uningested beads bound CAP accumulated region; yellow arrows, CAP accumulated live CHO pinching-off site at amoeba surface; blue arrows, CAP accumulated closure site at internal trogosome. **(B)** HA-PMCA expressing *E histolytica* trophozoites were co-incubated with human serum coated beads for 30 min (upper panels) or CellTracker Blue-stained live CHO cells for 30 min (lower panels). Cells were fixed and HA fusion protein and Atg8 were visualized with anti-HA and anti-Atg8 antibodies with Alexa Fluor 488 and 568 conjugated secondary antibodies, respectively. Black arrowheads, PMCA negative Atg8 positive phagosome/trogocytic cup; white arrowheads, PMCA and Atg8 double-positive phagosome/trogocytic cup; yellow arrowheads, PMCA positive Atg8 negative phagosome; white arrows, PMCA positive Atg8 negative trogosome. **(C)** Myc-STX expressing *E histolytica* trophozoites were co-incubated with human serum coated beads for 30 min (left panels) or CellTracker Blue-stained live CHO cells for 15 min (right panels). Cells were fixed and Myc fusion protein was visualized with an anti-Myc antibody with Alexa Fluor 488 conjugated secondary antibody. White arrowheads, STX positive phagosome/trogosome. **(D)** FLAG-Rab7G expressing *E histolytica* trophozoites were co-incubated with CellTracker Blue-stained live CHO cells for 15 min. Cells were fixed and the FLAG fusion protein was visualized with anti-FLAG antibody with Alexa Fluor 488 conjugated secondary antibody. Bars, 10 μm.

To validate the involvement of the proteins implicated by phagosome proteome analysis in the *atg8-*gs strain, we chose four representative proteins from the list of proteins that were less abundant in phagosomes isolated from the *atg8*-gs strain for subsequent immunolocalization studies (**Figure 3**). We selected these four proteins for the following reasons: (1) the candidates are not cargos such as lysosomal hydrolases; (2) they are presumed to be involved in phagosome/trogosome formation and/or maturation, but no studies had been previously conducted to demonstrate it. Adenylyl cyclase- associated protein (CAP, EHI_136150) was expected to be involved in phagosome formation *via* actin regulation. Furthermore, CAP was the most abundant protein detected in phagosomes from control only after two intermediate subunits of Gal/GalNAc specific lectin, Igl1 and Igl2. Plasma membrane calcium-transporting ATPase (PMCA, EHI_054830) was expected to be involved in calcium homeostasis but was never previously investigated as a phagosome-associated protein. SyntaxinB (STX, EHI_021410) was previously identified from *E. histolytica* phagosomes (Watanabe et al., 2020). Rab7G (EHI_187090) showed a sharp contrast in the differential presence between control and *atg8*-gs. Both STX and Rab7G are well established membrane traffic regulators but were never examined as potential Atg8-associated regulators of phagocytosis and trogocytosis.

Localization of CAP and Atg8 was examined in GFP-CAP expressing *E. histolytica* transformant after the trophozoites were coincubated with beads for 30 min. GFP-CAP was not localized to phagosomes that contained human serum-coated beads, as monitored by GFP fluorescence. The phagosomes were occasionally but rarely labeled with Atg8 (black arrowheads), detected by anti-Atg8 antiserum (**Figure 3A**, cell #1, upper panels). When GFP-CAP expressing trophozoites were incubated with live CHO cells to allow ingestion (**Figure 3A**, lower panels), GFP-CAP was accumulated at a very confined region of the closing trogosome, where pinching off of the target cell and fusion of two juxtaposed regions of the invaginated plasma membrane occur (**Figure 3B**, lower panels, cell #2 and #4, blue and yellow arrows). GFP-CAP was also concentrated in the region connecting the trogosome and the trogocytic tunnel, where the closure of the trogosome occurs (**Figure 3B**, cell #3, upper right trogosome, light blue arrows). Note that cell #3 shows two trogosomes, one Atg8-positive (completely enclosed, left bottom, indicated by black arrowheads) and one Atg8-negative (not enclosed, right top, yellow arrowheads) trogosomes, and only on Atg8-negative trogosome (right-top), the punctate GFP-CAP signal was observed on the pinch-off region of the unclosed (or closing) trogosome. Note that Atg8 is recruited to phagosomes at the very early phase (within 10 min) of internalization but dissociated soon after, based on the previous study (Picazarri et al., 2015).

Localization of PMCA and Atg8 was examined in HA-PMCA expressing *E. histolytica* transformant. HA-PMCA was distributed to the membrane of enclosed phagosomes containing beads. However, HA-PMCA was not localized to all phagosomes, but only about 26% of the phagosomes at 30 min of coincubation. HA-PMCA was mainly (about 80%) localized to the Atg8-negative bead-containing phagosomes (**Figure 3B**, top panels, cell #5-7, yellow filled arrowheads), and occasionally (about 20%) Atg8-positive phagosomes (white arrowheads). In contrast, when the amoebae were fed with CHO cells, Atg8, which is recruited to the trogosomes in the very early phase (~10 min) as described above, was localized to about 12% of trogosomes at 30 min, but rarely to HA-PMCA-positive trogosomes (**Figure 3B**, lower panels, black arrowheads). During trogocytosis, particularly in the early invagination phase, Atg8 was localized to the bottom of the trogocytic cup (i.e., unenclosed trogosome) (**Figure 3B**, bottom panels, black arrowheads in cell #8 and white arrowheads in cell #9), but HA-PMCA was only weakly and occasionally colocalized (only weak association in cell #9, white arrowheads, but not in cell #8, black arrowheads). After the trogosome was enclosed, Atg8 was dissociated and absent from the trogosome, and HA-PMCA was well associated with the entire membrane of the closed trogosomes (white arrows, cells #8 and #10). This observation is consistent with the model that the translocation of PMCA to phagosomes and trogosomes occurs in the later phase (after closure) of phagocytosis/trogocytosis.

Localization of EhSyntaxinB (STX) and Rab7G was examined in the transformant expressing Myc-STX (**Figure 3C**) or FLAG-Rab7G (**Figure 3D**). Myc-STX was found to be concentrated in confined regions on the phagosomes containing serum-coated beads (**Figure 3C**, left panels) and on the trogosomes containing live CHO cells (right panels). In contrast, Rab7G, detected by antibody against the Flag tag fused to the N-terminus, was not localized to either bead-containing phagosomes or CHO cell-containing trogosomes after internalization of prey (**Figure 3D**).

## Discussion

### Comparative phagosome proteomics of *atg8*-gs strain yielded a list of phagosome proteins that are either recruited to or excluded from phagosomes in an Atg8-dependent manner

In this study, we identified 127 and 107 proteins whose abundance in phagosomes is either decreased or increased by *atg8*-gene silencing, respectively. The proteins that were reduced in abundance by *atg8*-gene silencing are assumed to be transported to phagosomes *via* Atg8-regulated, or, if not directly regulated, -associated mechanisms. We previously reported that the *atg8*-gs strain showed a only slight (although not proven to be statistically significant) reduction in bacteria ingestion but remarked delay in acidification of phagosomes when compared to control (Picazarri et al., 2015). Thus, it was expected that Atg8 is mainly involved in acidification and maturation of phagosomes, but not in the initial internalization of prey per se. Thus, our new list of proteins that were reduced in abundance by *atg8* gene silencing largely represent the proteins whose phagosome recruitment is dependent upon Atg8. The list should provide baseline information on the proteins involved in phagosome maturation directly or indirectly regulated by Atg8. We estimate that approximately one-third (127 of 338) of phagosomal proteins are trafficked in Atg8-associated mechanisms. It is worth noting that no known Atg8 binding proteins such as AIR-containing proteins were found to be enriched by *atg8* gene silencing. If Atg8 on the phagosomal membrane regulates recruitment of Atg8 binding proteins in a spatiotemporal specific fashion, reduction in or loss of specific proteins in phagosomes would be expected by *atg8* gene silencing. Thus, the role of Atg8 may not be regulation of recruitment of specific binding proteins, but facilitation of phagosome maturation by hemifusion, fusion, deformation, and out-budding as suggested (Nakatogawa et al., 2007; Maruyama et al., 2021).

On the contrary, a list of proteins that were more abundant in phagosomes from the *atg8-*gs strain predominantly belong to two major categories related to ribosome functions and cytosolic metabolism. We had initially expected enrichment of proteins associated with the early phase events of phagocytosis, such as binding and internalization, actin cytoskeleton regulation, and signaling. This is because we assumed that *atg8* gene silencing may interfere with the recycling of surface receptor(s) that are internalized. We do not fully understand the physiological significance of the enrichment of ribosomal proteins but believe the discovery is meaningful, as phagosomes do not usually have physical contact with the ER although the phagosomal membrane is supplied from the ER (Gagnon et al., 2002). It is worth noting that one of the main components of the ER translocon complex, Sec61, which is known to mediate ER-ribosome interaction (Zimmermann et al., 2011), was 3.1-3.7 fold lower in phagosomes from *atg8*-gs compared to that from control (**Supplementary Table 4**). This observation may suggest that Atg8 may be involved in the recruitment of membrane components from the ER *via* interaction with ribosomes. The molecular basis of Atg8-dependent ribosome association to phagosomes needs to be elucidated in the future.

### Traffic of lysosomal hydrolase carriers (transport receptors) and hydrolases is Atg8-dependent

Our analysis by PANTHER protein class categorization (**Figure 1B**) showed that the top two functional classes of proteins that were reduced in abundance by *atg8* gene silencing are metabolite interconversion enzyme (PC00262, 21 proteins) and protein modifying enzyme (PC00260, 15 proteins). Among them, a variety of lysosomal enzymes and their known carriers were identified. They are cysteine protease binding protein family 6 (CPBF6, EHI_178470) and CPBF8 (EHI_059830), involved in the lysosomal targeting of hydrolases (**Supplementary Table 1**). CPBFs are a lineage-specific family of eleven members of hydrolase receptors/carriers with CPBF1 being the authentic cysteine protease binding protein/carrier, originally identified as EhCP-A5 binding protein (Nakada-Tsukui et al., 2012; Marumo et al., 2014; Nakada-Tsukui and Nozaki, 2015). While CPBF1 is involved in targeting of a panel of cysteine proteases including EhCP-A5 from the ER to lysosomes (Nakada-Tsukui et al., 2012), CPBF6 and CPBF8 carry α-amylase (EHI_023360), γ-amylase (EHI_044370) (for CPBF6), β-hexosaminidase (EHI_148130), and lysozymes (EHI_199110, EHI_096570) (for CPBF8), respectively (Furukawa et al., 2012; Furukawa et al., 2013). Atg8-dependent targeting was found for CPBF6 and CPBF8, but not for CPBF1. Lysosomal hydrolases shown to be reduced in abundance by *atg8* gene silencing include some CPBF ligands such as EhCP-A2 (EHI_033710, the ligand for CPBF1 and 4), EhCP-A5 (EHI_168240, the ligand for CPBF1), α-amylase (EHI_023360, the ligand for CPBF6 and 10), β-hexosaminidase (EHI_007330, the ligand of CPBF7), the uncharacterized protein which is previously annotated as γ-amylase (EHI_044370, the ligand of CPBF6) (**Supplementary Table 1**). These observations reinforce the notion that targeting of CPBFs is under the regulation of Atg8.

### Novel phagosomal proteins identified by differential phagosome proteomics by *atg8* gene silencing

We have identified for the first time a few important but uncharacterized proteins, such as CAP, PMCA, and STX, that likely play an important role in trogosome and phagosome biogenesis. We have validated their localization and colocalization (or lack of colocalization) with Atg8 by immuno fluorescence assay. These proteins were assumed to be recruited *via* an Atg8-dependent manner. CAP is identified as an adenylyl cyclase binding protein, and its role was initially assumed to be actin regulation (Ono, 2013; Zhou et al., 2014; Rust et al., 2020). CAP is a multifunctional protein containing six domains: oligomerization, helical folded, WASP homology 2, flox by proline-rich, CAP, and RP2 domains. CAP cooperates with its binding proteins, such as profilin, twinfilin, and actin binding protein-1, and regulates actin filament assembly and disassembly by its nucleotide exchange activity towards G-actin (Ono, 2013; Rust et al., 2020). We had expected that CAP might be localized on the phagocytic cup and the early phagosomal membrane. As expected, CAP was concentrated in a very confined region where a phagosome or a trogosome is enclosed. The image of CAP being concentrated at the pinch-off region (**Figure 3**, cell #3) resembles that of the division ring of oocytes and organelles such as chloroplasts and mitochondria (Yoshida et al., 2006; Imoto et al., 2018). These observations suggest that CAP in *E. histolytica* is involved in the closure and pinch-off of the phagosomes. In *E. histolytica*, calcium binding protein (CaBP)3, CaBP5, myosin IB, atypical kinase 1 (AK1), and two components of Arp2/3 complex, ARPC1, and ARPC2, have shown to be involved in phagosome closure during erythrophagocytosis (Babuta et al., 2020). It may be possible that CAP is cooperatively involved in the closure/pinch-off of the phago/trogosomes. It is worth mentioning that by gene survey, *E. histolytica* lacks typical membrane-associated adenylyl cyclase and has soluble adenylyl cyclase (Agarwal et al., 2020). Agarwal and colleagues have shown an adenylyl cyclase activator or inhibitor respectively increases or decreases erythrophagocytosis in *E. histolytica* (Agarwal et al., 2020). The role of cAMP signaling and CAP in actin regulation during phagocytosis/trogocytosis needs to be elucidated in *E. histolytica* in future. Although CAP was previously identified in phagosomes of macrophages (Garin et al., 2001; Oliveira et al., 2012), its localization during phagocytosis was not examined (Ono, 2013; Rust et al., 2020). In *Dictyostelium*, CAP was found on the phagocytic cup (Sultana et al., 2009); however, it remains to be determined whether CAP localizes to the pinch-off region of trogosomes during trogocytosis.

The *E. histolytica* genome encodes five calcium-ATPases, three of which are PMCAs (EHI_016480, EHI_030830, EHI_054830), and two encode organelle ATPases [sarco/endoplasmic reticulum Ca^2+^-ATPases (SERCA), EHI_027710 and Golgi Ca^2+^-ATPases (SPCA/PMR1), EHI_065670] (Martinez-Higuera et al., 2013). PMCAs are known in general to be localized on the plasma membrane (Chen et al., 2020), We have shown that in *E. histolytica*, PMCA (EHI_054830) is distributed to internal vesicles, and importantly, translocated to phagosomes at the late stage of phago- and trogocytosis (**Figure 3B**). Thus, it is assumed that the PMCA is recruited from vesicles to phagosomes in an Atg8-dependent manner. Among the five Ca^2+^-ATPases, four of them, except for EHI_027710, are listed in 338 phagosomal proteins. Also, two calcium-transporting ATPases were reported in our previous phagosome proteome study (Okada et al., 2005). PMCA and SERCA were also identified from phagosomes of macrophages (Rogers and Foster, 2007; Shui et al., 2008). However, their role in phagocytosis or phagosome maturation was not analyzed. Based on the predicted topology, in *E. histolytica*, PMCA on the phagosome likely transports cytosolic calcium into phagosomes to reduce cytosolic calcium concentrations (Chen et al., 2020).

We have also shown that one of the syntaxins, SyntaxinB (STX, EHI_021410), is trafficked to phagosomes in an Atg8-dependent manner, which was confirmed by immunolocalization of myc-STX to phagosomes and trogosomes (**Figure 3D**). Syntaxin belongs to the soluble N-ethylmaleimide-sensitive fusion factor attachment protein (SNAP)-receptors (SNAREs) and is involved in membrane fusion (Kloepper et al., 2007; Koike and Jahn, 2022). The *E. histolytica* genome has 25 SNAREs including at least three plasma membrane, two TGN/lysosome, and one cis-Golgi localized SNAREs, as predicted by phylogenetic analyses, although the localization of other SNAREs remains undetermined (Saito-Nakano et al., 2021a). STX found in this study is grouped into a clade that is unique to *E. histolytica* among the representative SNAREs from *Homo sapiens*, *Arabidopsis thaliana*, and *Saccharomyces cerevisiae*; thus its function is unpredictable (Saito-Nakano et al., 2021a). Importantly, only one SNARE, out of 25, is included in a repertoire of 338 phagosomal proteins, strongly suggesting that STX is the predominant SNARE involved in phagosome biogenesis. As shown in **Figure 3D**, the localization of Myc-STX is on internal vesicles, not on the plasma membrane, suggesting that STX is localized to either the ER or endosomes. Translocation of ER-residing SNAREs to phagosomes was demonstrated in macrophages (Hatsuzawa et al., 2006) and endosomal SNAREs were detected from macrophage phagosomes (Garin et al., 2001). It is conceivable that *E. histolytica* STX is involved in the recruitment of the ER to phagosomes to provide membranes and membrane-associated constituents or in phagosome-lysosome fusion during phagosome maturation. It is necessary to investigate the partner SNAREs that make a complex with STX to understand organelle specificity.

### Rab7D and Rab7G may be involved in prephagosomal biogenesis

Recruitment of Rab7D and Rab7G to phagosomes was suggested to be regulated by Atg8, as demonstrated in our *atg8-*gs phagosome proteomic analysis. Among nine Rab7 isotypes, Rab7D and Rab7G share unique features, i.e., amino acid substitutions in the inter-switch region and the acidic amino acid stretch at the carboxyl terminus, and are expected to have similar roles and/or regulation (Saito-Nakano et al., 2021b). Rab7D was detected in previous phagosome proteomic studies (summarized in **Supplementary Table 7**) (Marion et al., 2005; Okada et al., 2005; Boettner et al., 2008; Watanabe et al., 2020). Rab7D is localized to prephagosomal vacuole (PPV) and involved in its maturation (Saito-Nakano et al., 2021b). PPV appears to be a unique compartment to *E. histolytica*, likely involved in phagosome/trogosome biogenesis as a preparatory compartment. Its formation is induced by attachment with nucleated mammalian cells and erythrocytes (Saito-Nakano et al., 2004). PPV is first associated with Rab5 and Rab7A upon formation (~10 min), and Rab5 is subsequently dissociated from PPV (at ~30 min). PPV then fuses with the bait-containing phagosome to deliver the content (e.g., hydrolases) in PPV to the phagosome. Rab7D is transiently recruited to PPV after dissociation of Rab5 and before or together with Rab7B. Rab7G may be localized on PPV in a similar fashion to Rab7D, although recruitment of Rab7G was not demonstrated in this study. However, we cannot exclude the possibility that PPVs were pulled down with phagosomes *via* interaction between PPVs and phagosomes. In this case, Rab7G may contribute to phagosome maturation *via* the regulation of PPVs. Although Rab7A, Rab7B, and Rab7D were shown to be associated with PPV by imaging analysis (Saito-Nakano et al., 2004; Saito-Nakano et al., 2007; Saito-Nakano et al., 2021b), *atg8* gene silencing seems to influence the abundance of only Rab7D, but not Rab7A or Rab7B, on phagosomes. Thus, Atg8 unlikely affects tethering/fusion of PPV with the phagosome, but principally affects PPV maturation.

### A possible role of Atg8 in phagosome maturation in *E. histolytica*

It is worth rephrasing that early recruitment of Atg8 before the closure of the phagosome/trogosome in *E. histolytica* is a unique feature that is markedly different from mammalian cells. It was shown in mammalian cells that LC3 is recruited to LC3-associated phagocytosis (LAP) after the closure of the phagosome and depending on the generation of phosphatidylinositol 3-phosphate (PI3P) and reactive oxygen species (ROS) (Martinez et al., 2015). In contrast, our preliminary observation suggests that Atg8 recruitment to the phagocytic cup and the phagosome proceeds that of PI3P (Nakada-Tsukui, unpublished). As discussed above, Atg8 is likely involved in phagosome maturation *via* its tethering/hemifusion activity. Spatiotemporal regulation of Atg8 recruitment to the nascent phagosomes must play a key role in the phagosome maturation process (Marat and Haucke, 2016). PI3P is present on the nascent newly-formed phagocytic cup (Powell et al., 2006; Nakada-Tsukui et al., 2009). Also, it is known that PI3P binding proteins are involved in the recruitment of the Atg5-12/16 complex at the site of LAP (Dooley et al., 2014; Martinez et al., 2015). Currently, it is well accepted that Atg8 is involved in a broad range of degradation processes other than autophagy. There are still quite many key questions such as Atg8 targeting mechanisms, the role of Atg8 on phagosome/trogosome maturation, and the involvement of calcium in Atg8 recruitment, which must be addressed in the future.

## Data availability statement

The datasets presented in this study can be found in online repositories. The names of the repository/repositories and accession number(s) can be found in the article/**Supplementary Material**.

## Author contributions

Conception or design of the work, KN-T. Data collection, KN-T, NW, KS, RW, and EM. Drafting the article, K-NT and TN. Critical revision of the article, KN-T and TN. Final approval of the version to be published, KN-T, NW, KS, RW, EM, and TN.

## Funding

This research was funded by Grants-in-Aid for Scientific Research (B) (KAKENHI JP18H02650 and JP21H02723 to TN), Scientific Research (B) and Scientific Research on Innovative Areas (JP19H03463 and JP20H05353 to KN-T), Promotion of Joint International Research (Fostering Joint International Research (B)) (21KK0139 to KN-T) from Ministry of Education, Culture, Sports, Science and Technology (MEXT) or Japan Society for Promotion of Sciences (JSPS), Grant for research on emerging and re-emerging infectious diseases from Japan Agency for Medical Research and Development (AMED, JP20fk0108138 to TN; JP20fk0108139 to KN-T), and Grant for Science and Technology Research Partnership for Sustainable Development (SATREPS) from AMED and Japan International Cooperation Agency (JICA) (JP20jm0110022 to TN).

## Acknowledgments

We thank Dr. Nicholas E. Sherman, W.M. Keck Biomedical Mass Spectrometry Laboratory, University of Virginia, USA for mass spectrometric analysis.

## Conflict of interest

The authors declare that the research was conducted in the absence of any commercial or financial relationships that could be construed as a potential conflict of interest.

## Publisher’s note

All claims expressed in this article are solely those of the authors and do not necessarily represent those of their affiliated organizations, or those of the publisher, the editors and the reviewers. Any product that may be evaluated in this article, or claim that may be made by its manufacturer, is not guaranteed or endorsed by the publisher.

## Supplementary material

Supplementary Figure 1 Protein class distribution of phagosomal proteins in mock control and *atg8*-gs strain. Phagosomal proteins of indicated strains were analyzed for their protein class by PANTHER and percentage distribution was shown in the pie graph.
