# Supplementary figures and images for: Proteomic analysis of Atg8-dependent recruitment of phagosomal proteins in the enteric protozoan parasite Entamoeba histolytica
Source: Front Cell Infect Microbiol. 2022 Oct 3;12:961645. doi: 10.3389/fcimb.2022.961645 (PMC9575557; doi:10.3389/fcimb.2022.961645)

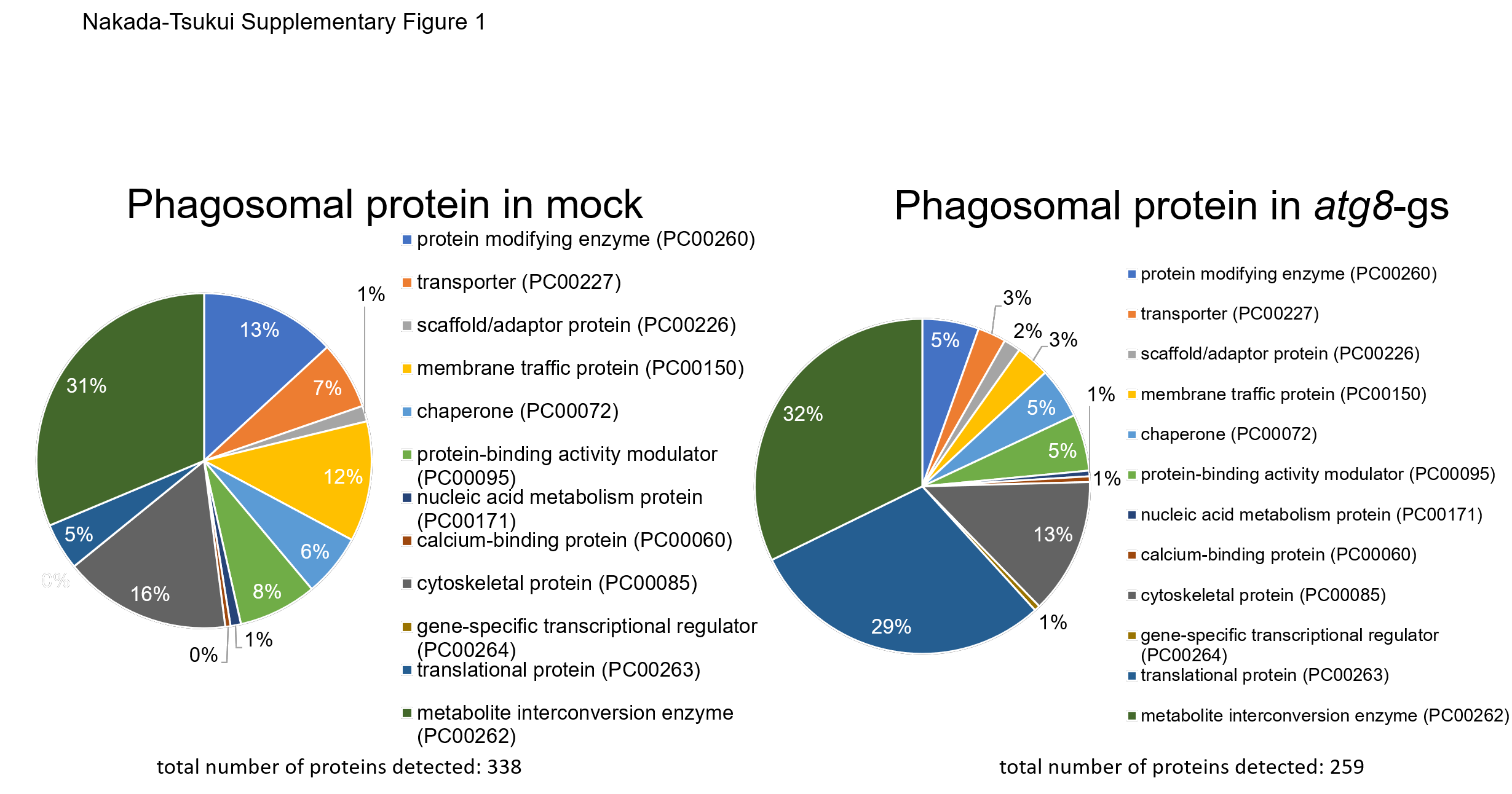

Supplement: Supplementary Figure 1 — Protein class distribution of phagosomal proteins in mock control and atg8-gs strain. Phagosomal proteins of indicated strains were analyzed for their protein class by PANTHER and percentage distribution was shown in the pie graph. [file Image_1.tif]
